# Evaluating the Taste Masking Ability of Two Novel Dispersible Tablet Platforms Containing Zinc Sulfate and Paracetamol Reconstituted in a Breast Milk Substitute

**DOI:** 10.3390/pharmaceutics14020420

**Published:** 2022-02-15

**Authors:** Samuel Orubu, Richard A. Kendall, Yucheng Sheng, Catherine Tuleu

**Affiliations:** 1 Department of Biomedical Engineering, College of Engineering, Boston University, Boston, MA 02215, USA; 2 Department of Pharmaceutics, UCL School of Pharmacy, University College London, 29-39 Brunswick Square, London WC1N 1AX, UK; shengyc@gmail.com (Y.S.); richardakendall@gmail.com (R.A.K.)

**Keywords:** taste, aversion, palatability, grittiness, neonates, milk, rapidly-disintegrating formulation

## Abstract

Milk is often used as a dispersion medium for medicines administration in young children but its taste-masking ability is unknown. A human taste panel was conducted to assess the potential of infant formula milk (Aptamil^®^ 1) to mask the taste of two model WHO priority medicines, zinc sulfate and paracetamol, manufactured as dispersible tablets. Simultaneously, the palatability of powder blends of the tablet platforms was assessed. Twenty healthy adult volunteers performed a swirl-and-spit assessment of placebos and API-containing blends in either a lactose-based or a mannitol-based dispersible tablet platform, reconstituted in 10 mL of either water or Aptamil^®^ 1. Eighteen samples were rated for aversion using a 100-mm Visual Analogue Scale, grittiness using a 5-point Likert scale, and “acceptability-as-a-medicine” evaluated as: “Would you find this sample acceptable to swallow as a medicine?” with binary answers of Yes/No. The API-containing formulations were more aversive than the placebos; the paracetamol-containing samples being more aversive than zinc sulfate samples. The platforms themselves were not aversive. Non-gritty samples had four-fold greater odds of being acceptable as a medicine. Aptamil^®^ 1 masked the taste of zinc sulfate in the mannitol-based formulation but did not mask the taste of paracetamol in either platform, suggesting a limited taste-masking ability, which may be API and formulation dependent.

## 1. Introduction

The development of acceptable, age-appropriate, medicines for children is challenging from both a pharmaceutical and clinical perspective [1]. Very young children (especially neonates 0–28 days) are a particularly challenging patient sub-population to address due to issues including swallowability, taste aversion and the need for frequent dose modifications [2]. There are also issues related to the safety of excipients, drug product stability on storage or during use in settings without access to refrigeration, and affordability of existing medicines [3,4,5,6]. These continuing challenges perpetuate the current need for better formulated medicines, as well as analytical tools to facilitate their development, for paediatric patients, in early development through to global manufacture and supply.

In 2008, the World Health Organization (WHO) proposed flexible solid dosage forms, for example dispersible tablets, as the preferred formulations for young children (0–5-year-olds) [7], who usually cannot safely swallow conventional solid oral dosage forms of sizes about 8–10 mm in diameter [8,9]. These medicines manufactured as solids are potentially more stable than liquids, especially in countries with hot and humid conditions where limited access to refrigeration exists. Flexible solid dosage forms can be taken intact, as a portion of a scored tablet, or immediately following suspension in water or (breast) milk at the point of administration, with or without the use of a medical device [7]. A key advantage of flexible solid dosage platforms is that they can be inexpensively made by established and well-understood compression technologies, and can be utilized as a modality for the low-cost local production of more easily swallowable, or age-appropriate, essential medicines for children in resource-limited settings, thus improving patient access to essential medicines [10].

Ideally, from a clinical development perspective, a simple formulation that is quick to develop and appropriate for early trials in small numbers of paediatric patients, in order to minimise formulation bridging risk as paediatric clinical studies progress, is desirable; as are the following: easily scaled-up and globally accessible (plug and play/simple platform technology); cheap to manufacture and likely to have good drug product stability. Using a target product profile approach based on the requirements of the flexible solid dosage form proposal, compendial specifications for a dispersible tablet and manufacturability, such a platform was developed [11]. Since studies in youngest children are going to be the last to start, from a drug development perspective using platforms such as the one described presently for this palatability assessment is easy to work back from.

Palatability is a derived organoleptic property comprised of flavour, smell, sight, taste, texture (grittiness or mouthfeel) and after-taste of a formulation and is a composite of both the active pharmaceutical ingredient (API) and excipients [12]. Palatability is, therefore, an important attribute to consider when developing new medicines for children, as it can affect overall acceptability [13,14]. With very young children, palatability is paramount to adherence and effective clinical outcomes [15]. The predominant taste of many medicines currently on the market is that of bitterness. For example, out of the 127 oral APIs on the WHO Essential Medicines List for children, 2011, for which taste information could be found, only 11 had a non-aversive taste [11]. Specific examples of bitter tasting APIs are: quinine hydrochloride, sildenafil citrate, caffeine citrate, isoniazid, telbivudine and paracetamol [16]. While this problem is relatively easily mitigated in the adult population, where the majority of patients do not suffer dysphagia, by coating a tablet with a taste-masking polymer, bitter tasting APIs for paediatric clinical use have generally been formulated with pharmaceutically acceptable sweetening and flavouring excipients to enhance the palatability of liquid dosage forms. Some of these functional excipients, however, suffer from issues of tolerability in certain paediatric age-groups, homogeneity of incorporation in the solid state, batch-to-batch variability and changes in functionality upon storage and safety [5,6,17,18] all of which can limit their use in paediatric formulations. Milk, as well as being a delivery or reconstitution vehicle, might be a useful modality for improving the taste of medicines for children. As a natural product, it might also have less of a safety concern. The use of milk or infant formula in the formulation, or administration, of medicines is established in the literature [19,20,21,22]. However, there are limited studies, to date, on the use of milk to improve palatability. 

The aim of this work, as part of a broader project, was to assess the ability of infant formula milk (Aptamil^®^ 1—see supplementary notes no. 1, S1), a breast milk substitute suitable from birth, to mask the taste of two model APIs. The objectives were to: (i) assess the palatability of two novel dispersible tablet platforms suitable for direct compression production, (ii) determine the contribution of taste and grittiness to palatability, (iii) assess the acceptability of these platforms as medicines, (iv) assess the (inherent) ability of infant formula milk to mask the taste of these platforms containing two model APIs. In that respect, a human taste panel reported the quality and intensity of the test stimuli (taste and mouthfeel of the samples) in comparison to water. In line with the WHO flexible solid dosage forms proposal, two novel and rapidly disintegrating tablet platforms containing Generally Recognized As Safe (GRAS) ingredients, both targeted to meet the British Pharmacopeial specifications for dispersible tablets of disintegration times < 3 min, fineness of dispersion < 710 micrometres [23], and of acceptable friability of less than or about 1% were developed [11]. The two formulation blends developed were given the names T1 (lactose-based) and T2 (mannitol-based) as described in Table 1 and were visually checked for rapid suspendability in both water and commercially available infant formula milk (Aptamil^®^ 1). The two model drugs selected for investigation were paracetamol and zinc sulfate, both of which are classified as essential medicines needed for a basic health-care system, i.e., those deemed to be efficacious, safe and cost-effective medicines for the treatment of priority conditions [24]. Paracetamol was chosen as it is well tolerated, established and is a widely available analgesic and antipyretic commonly used in children as young as 3 months of age. Zinc sulfate was chosen as it is commonly prescribed as an adjunct to oral rehydration therapy for the treatment of diarrhoeal disease, one of the leading causes of death globally with a mortality in 2016 of 446,000 in children under 5 years [25]. Both paracetamol and zinc sulfate were used at the maximum dose indicated for a 3-month-old child, and a volume of 10 mL of either water or infant formula milk was selected to represent a concentration of API dispersion close to the worst-case scenario that would be administered in practice [26].

## 2. Materials and Methods

### 2.1. Materials

Paracetamol and zinc sulfate, pharmaceutical grades (Fagron, Newcastle, UK), microcrystalline cellulose (Avicel^®^ PH101, FMC Biopolymer, Girvan, UK), crospovidone (Polyplasdone^®^ XL, FMC Biopolymer, Girvan, UK), sodium stearyl fumarate (FMC Biopolymer, Girvan, UK), lactose (SuperTab 14SD), sodium starch glycollate (Explotab CLV, Mendell GmbH, Volklingen, Germany); sodium croscarmellose (Ac-Di-Sol, FMC Biopolymer, Girvan, UK; mannitol, (Pearlitol^®^ 200SD Roquette, UK), magnesium stearate, technical grade (Sigma Aldrich, Dorset, UK); Buxton^®^ bottled water, Buxton, UK; Aptamil^®^ 1 first milk, ready-to-feed (Nutricia, Trowbridge, UK).

### 2.2. Preparation of Test Samples

In accordance with the British Pharmacopoeia (BP) general monograph, the production of the test blends was undertaken by competent staff and prepared extemporaneously under the supervision of a UK-registered pharmacist. Samples were prepared under strict quality measures in a dedicated area according to locally approved standard operating procedures (SOP). Here, the SOP refers to a set of safety checks in place at UCL, the study location, for studies of this nature to assure safety of participants.

For each bulk blend, a “sandwich” technique was used where the API, if present, was introduced to the mixing vial between two layers of excipients. Blends were prepared by mixing the required amounts of ingredients, excluding the lubricant (either magnesium stearate or sodium stearyl fumarate) in a Turbula^®^ mixer for 15 min at 32 rpm. Lubricant was then added and mixing continued for a further 5 min. After mixing, the blend was then passed through a sieve with a nominal aperture of 500 µm [S2]. Batch sizes were 100 g. The mixing vessel was a clean amber glass jar; the level of fill of the jar was between 40 and 70% and calculated based on the bulk densities of the individual blend components. On completion of blending, 330 mg of each of the blend listed in Table 1 was accurately weighed and transferred to a 30 mL universal tube, and labelled with a unique and randomised 3-digit code containing at least one number and one letter.

### 2.3. Testing Protocol

Participants/volunteers were recruited from undergraduate and postgraduate student cohorts at UCL, all of whom were young healthy adults between 18 and 40 years of age and able to understand English. The use of adult human panels is justified on the grounds of ethical concerns with the direct use of children as taste panels, reliability of outputs, and precedence [S5, S6].

Exclusion criteria were disorders of sense of taste or smell, drug or excipient allergies (including milk), lactose intolerance or any occurrence of dental care or medical treatment (with the exception of oral contraceptives) during the 15 days before study commencement. All volunteers were advised on all aspects of the study, including data confidentiality, both in writing and in person prior to agreement to participate in the study.

The protocol design (UCL Ethics Project ID Number 4612/006) was a single centre clinical study. Twenty volunteers completed two sessions of 2 hours’ duration over two separate days to reduce the burden on the participants, with at least a 48-h washout period in between sessions. Each volunteer evaluated nine formulations in each of the sessions, after receiving a training/calibration sample of spring (non-carbonated bottled) water. Participants were blinded to the composition of each sample. A sample size of 20 participants was used, which was considered sufficient to perform a statistical analysis to detect the differences among the dispersions from the inter-subject variabilities [S7]. Breakfast or a neutral lunch (not spiced, lightly salted) was permitted at least 30 min prior to investigation; volunteers who were smokers were precluded from smoking for at least one hour before, and during, the tests. Participants were visually isolated from each other by using movable screening boards to avoid any potential interaction and bias. A quiet environment that was light and airy was maintained to avoid any distraction.

Immediately prior to evaluation by the volunteers, the dry blends were reconstituted in 10 mL of water or infant formula milk by 10 s of manual shaking until visually homogenous. Following reconstitution, the zinc and paracetamol formulations contained a theoretical concentration of 2 mg/mL and 12 mg/mL of active ingredient, respectively. A total of 20 samples were prepared for each API formulation. One sample of each was presented to the participants for testing on two different days in a randomised order each day. The paracetamol formulations were tested on day 1 and the zinc on day 2.

The volunteers were instructed on each day to “swirl and spit” each sample to evaluate the palatability, whereby the test samples were swirled around the mouth for a period of 5 s, to ensure contact of the test sample with the oral surfaces inside the mouth, prior to ratings. Immediately upon expectoration, the participants rated the degree of aversion and grittiness using a computerised questionnaire with continuous and categorical scales, respectively. Before and after each sample, participants rinsed their mouths with water and consumed, if needed, plain crackers to neutralise the aftertaste of the previous sample prior to evaluating the next randomised sample 10 min later.

### 2.4. Assessments of Taste, Grittiness, “Acceptability as a Medicine” and Taste-Masking

Taste was assessed as the degree of aversion, recorded as the rated taste intensity or score, using the online questionnaire software Qualtrics^®^ (http://www.qualtrics.com) on a 10 cm visual analogue scale with anchors of “not aversive” and “very aversive” (left to right) at either end. The scale had a sliding pointer that participants moved towards each anchor depending on their perception of the taste of the presented formulation. Prior to evaluation of the test samples, water was used as a negative control to calibrate the taste response of each of the 20 volunteers; each participant was instructed to enter an aversion value of 0 for the water. Thereafter, each of the reconstituted suspensions was evaluated as described and an aversion value assigned, with reference to water. Readings on the scale were automatically measured and recorded (in a separate programme not visible to the participant) on a 100 mm scale from 0 for “not aversive” to 100 for “very aversive”. The pointer was set such that participants would only be able to rate the sample either as not aversive (0–49.9) or aversive (50.1–100) on the scale. Grittiness was assessed for each sample using a 5-point Likert scale from “not gritty” (1) to “gritty” (5). Samples rated 1–3 were regarded as not gritty, and 4–5 as gritty. “Acceptability as a medicine” was assessed as a response to the question: “Would you find this sample acceptable to swallow as a medicine?” The responses were either “yes” or “no”.

Taste-masking was assessed as the ability of a formulation dispersed in infant formula milk to have a significantly reduced taste score compared to the same formulation dispersed in water. The two platforms were compared with respect to zinc and paracetamol. In addition to these numerical, categorical and binary responses, participants were also given the option to provide additional qualitative written comments relating to their organoleptic perception of each sample.

Using the collected data, the contributions of the measured variables of taste and grittiness to “acceptability as a medicine” was then analysed.

### 2.5. Analysis of Results

Statistical analysis of the questionnaire data was performed using SPSS 26 (IBM SPSS). The related-samples Wilcoxon signed-rank test was employed to test differences in mean scores for taste and taste-masking between dispersions in water and infant formula milk.

The contributions, and association, of taste and grittiness to “acceptability as a medicine” were analysed by both descriptive statistics using participants’ mean taste scores for samples stratified into those deemed “unacceptable as a medicine” and those deemed “acceptable as a medicine”, as well as by a regression analysis. In the descriptive analysis, taste scores for “unacceptable as a medicine” formulations were expressed in terms of a minimum aversion value, and for “acceptable as a medicine” in terms of a maximum aversion score. To assess the relative contributions of taste and grittiness to acceptability, a binary logistic regression analysis was performed using SPSS 26 (IBM SPSS). Grittiness was recoded into two categories of not gritty (grittiness scores of 1–3) and gritty (grittiness scores of 4 and 5).

## 3. Results

Each of the 20 volunteers attended all of the sessions as planned. There were no refusals, or outward signs of physical discomfort in any of the volunteers, and no adverse effects were recorded. 

### 3.1. Overall Assessments of Taste, Grittiness and “Acceptability as a Medicine”

#### 3.1.1. Taste

**Figure 1:**
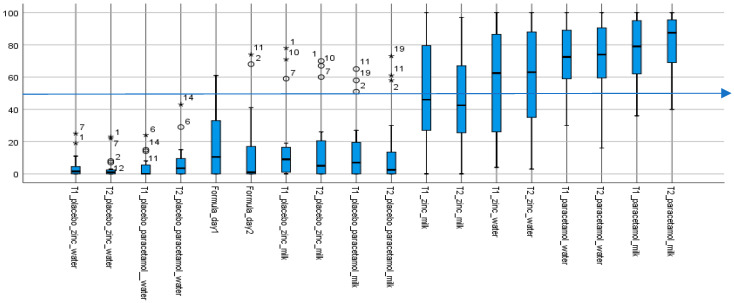
Summary of aversion scores for all 18 samples (except water). Aversion (taste) scores of test formulations. The box plots show the median taste score and ranges; outliers are indicated by the circles and extremes by the stars; with the numbers being volunteers’ de-identified IDs. (n = 18; as water score, assigned an anchored aversion of 0, on both days were excluded from the chart). T1 and T2, respectively lactose and mannitol, are the two different rapidly-disintegrating tablet platforms. All placebos, including infant formula milk (Formula_day1 and Formula_day2) with median scores below 50 (indicated by the blue line at 50) were not aversive. In contrast, with median taste scores above 50, all API-containing formulations were aversive, except for the two that contained zinc in infant formula milk. The paracetamol formulations, with interquartile ranges above 50 were the more aversive.

Taste scores for the 18 samples evaluated against water, which was assigned an anchored aversion score of zero, ranged from low or “not aversive” (or 0–49.9) for placebos to high or “aversive” (or 50.1–100) for API-containing formulations, with varying degrees of individual variability (Figure 1). 

The aqueous placebo formulations had the lowest median taste scores and variability, followed by infant formula milk and associated placebos with similarly low taste scores but greater variabilities. With the four aqueous placebos, all taste scores—medians and “maximums”—were <20, and <50 with outliers, values between 1.5 and 3× the “maximum” and extremes, values above 3× the “maximum”, included. Taste scores (medians and “maximums”) for the six infant formula milk and associated placebos, while mostly less than, or around, 40, had outliers with taste scores >50. The exception was the infant formula milk on day 1 (Formula_day1) with a maximum taste score >50 which, while it had no outliers, had the greatest variability as indicated by the range of values. Outliers and extremes were the same specific individuals—six volunteers #1, 2, 7, 9, 10 and 11, mostly 2 and 11, and frequently in sets of two or three. Together, the infant formula milk samples—the formula milk itself and associated platform dispersions—had the greatest variability in taste scores among all three sets of 10 placebo samples, including the presence of outliers and extremes who found them to be aversive.

In contrast, the eight API-containing formulations were all aversive, with median taste scores > 50, apart from the zinc formulations in infant formula milk in both the lactose (T1) and mannitol (T2) platforms, which had median scores < 50. In general, taste scores were more distributed for the zinc containing samples. While zinc had lower median taste scores with interquartile values spanning both aversive and non-aversive ranges, paracetamol samples, in contrast, were skewed towards the aversive scale.

Thus, while the formulations without an API were assessed by the majority as “not aversive” (median taste scores <50), the API-containing formulations were “aversive” (median taste scores >50), apart from the zinc formulations in milk.

#### 3.1.2. Grittiness

**Figure 2:**
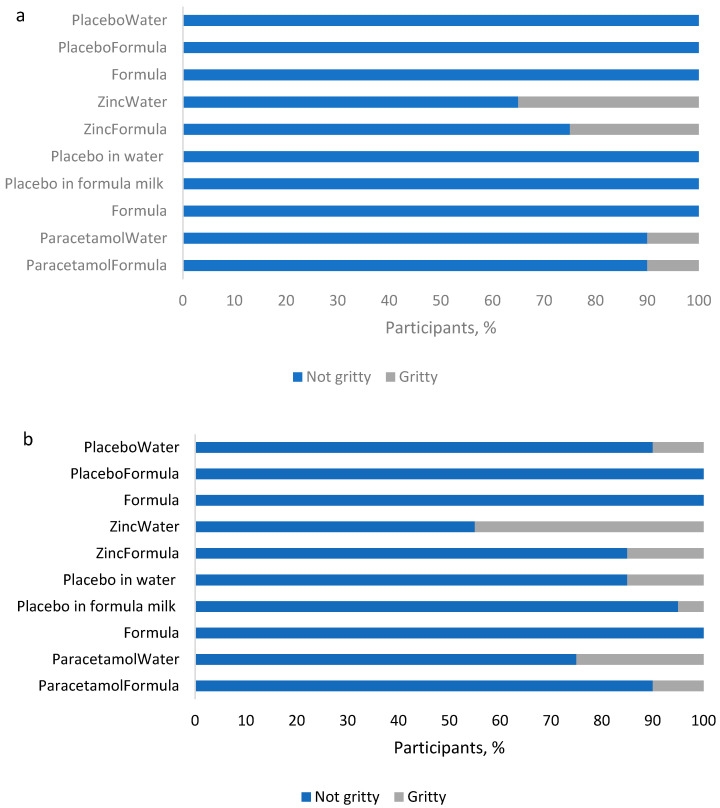
Grittiness of samples in the two platform formulations: (**a**) T1 (lactose), and (**b**) T2 (mannitol).

The grittiness of samples depended on both platform and API (Figure 2). 

Platforms differed in terms of perceived grittiness of samples, with more of the mannitol-based samples assessed as gritty (70%, 7/10) compared to the lactose-based samples (40%, 4/10). While the mannitol-based samples assessed as gritty included placebos without APIs, the lactose-based samples rated gritty were only those with included APIs. However, with mannitol, only a maximum of three volunteers found the placebo dispersion in water to be gritty.

Of the two APIs, zinc sulfate samples were assessed as gritty by more volunteers, with the zinc in water dispersion rated gritty by 35% of volunteers when in the lactose platform and by 45% when in the mannitol platform. In comparison, paracetamol samples were rated gritty by only 10% of volunteers, except for the dispersion in water with the mannitol formation, which was rated gritty by 25%. Thus, overall, while a greater proportion (≥55%) of volunteers rated each sample as not gritty, there were platform and sample specific differences.

#### 3.1.3. Acceptability

**Figure 3:**
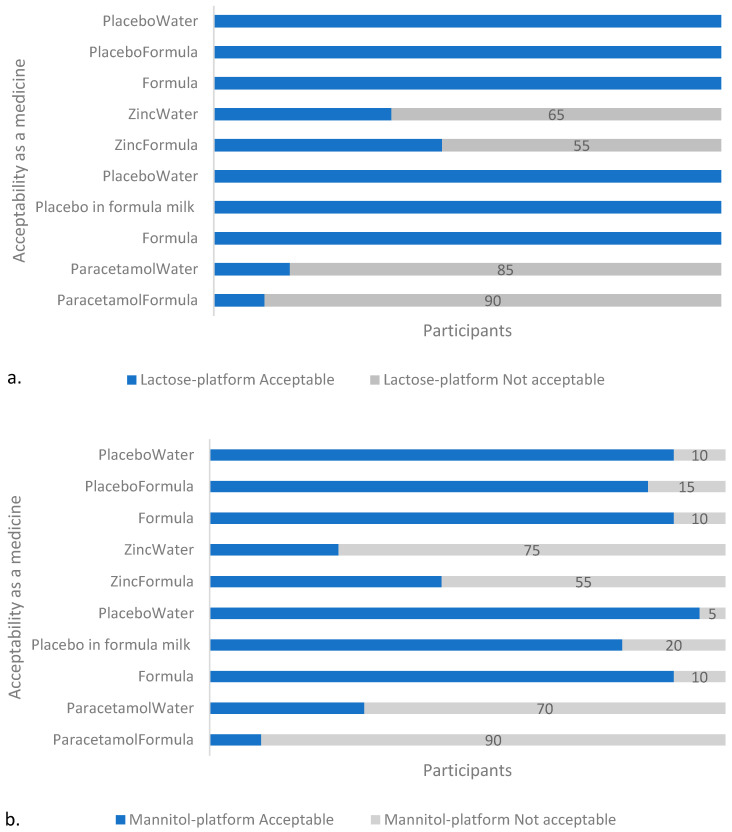
Acceptability of samples in: (**a**) T1 (lactose), and (**b**) T2 (mannitol) platform. With both platforms, the API-containing samples were assessed by the majority (≥55%, n = 20) of participants as unacceptable as a medicine, with the paracetamol-containing formulations unacceptable to the greater majority (≥70%, n = 20). Note: Formula and PlaceboWater appears twice on the y-axis to account for the two separate sessions (on two different days) with the two different APIs per platform in which they were used as placebos and reference samples, respectively.

The API-containing formulations were less “acceptable as a medicine” than the placebos or infant formula milk, regardless of platform (Figure 3). The formula milk control was well tolerated by the cohort of volunteers with 85% (17/20) of volunteers describing the taste as acceptable during both of the sessions. One volunteer consistently described the taste as “not acceptable” during each of the two tasting sessions, and two other volunteers each described the taste as unacceptable during 1 of the 2 sessions: one on the first day, and the other on the second, which does not imply that the cause of the perceived poor palatability was due to a factor other than intra-individual variability.

### 3.2. Taste-Masking Ability

The lactose (T1) and mannitol (T2) platforms differed in the taste-masking ability of infant formula milk with zinc but not paracetamol (Table 2). With the mannitol (T2) platform, the zinc formulation in infant formula milk had a significantly lower mean taste score than in water (*p* = 0.02); however, this difference was not observed with paracetamol (*p* = 0.12). In contrast, for the lactose (T1) platform, the zinc formulation in infant formula milk did not differ from that in water (*p* = 0.26), and this was also the case with the paracetamol formulations (*p* = 0.34). 

### 3.3. Contributions of Taste and Grittiness to the Acceptability of Samples

**Figure 4:**
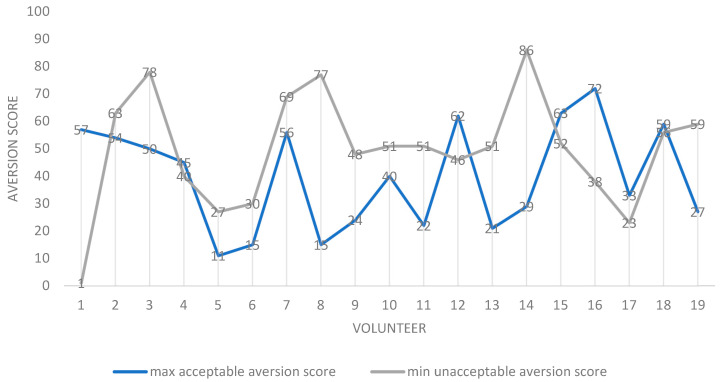
Scatter plot of maximum “acceptable-as-a-medicine” and minimum “unacceptable-as-a-medicine” aversion scores for all volunteers (participants). For each participant, the values are the mean for all 20 formulations assessed. Notes: 1. N = 19 as one volunteer was discounted as all samples were rated as acceptable as a medicine. 2. Volunteer 1 appears to be an outlier, as a sample rated as having an aversion value of 1 was described as being unacceptable as a medicine. This sample was also recorded as being “not gritty”. 3. In seven volunteers, contrary to expectation, the maximum “acceptable-as-a-medicine” taste scores were greater than the minimum “unacceptable-as-a-medicine” aversion scores: 1, 4, 12, 15, 16, 17 and 18.

The contributions of taste and grittiness to the acceptability of samples were assessed by two approaches. The first approach used a “qualitative” reading of average aversion scores. Here, the “overlap” between aversion and grittiness was used to infer relative contributions. Overall, across participants, the average minimum aversion value for a formulation deemed “unacceptable as a medicine” (52.3) was greater than the average maximum value for a sample deemed to be “acceptable as a medicine” (30.6). This accords with the pre-set values, and was expected. However, when individual scores were considered, for the majority of volunteers (13/20), the maximum aversion score for a sample deemed “acceptable as a medicine” was lower than the minimum aversion score for a sample deemed “unacceptable as a medicine” (Figure 4), leaving a minority for which this was not true. 

**Figure 5:**
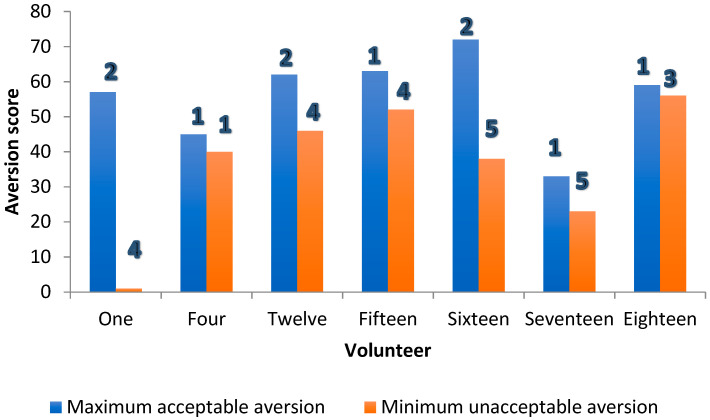
Threshold aversion (bars) and grittiness scores (numbers 1–5) for formulations in seven volunteers where the expected difference in mean individual taste scores between “acceptable-as-a-medicine” and “unacceptable-as-a-medicine” aversion score was not observed.

Where this was not true for the minority (7/20), and the maximum “acceptable-as-a medicine” score exceeded the minimum “unacceptable-as-a-medicine” aversion score, on six out of these seven occasions, the grittiness for the minimum “unacceptable-as-a-medicine” aversion score exceeded that at the maximum “acceptable-as-a-medicine” aversion score by at least two grittiness units (Figure 5).

This was suggestive of grittiness making a significant contribution to the overall perception of acceptability as a medicine.

**Figure 6:**
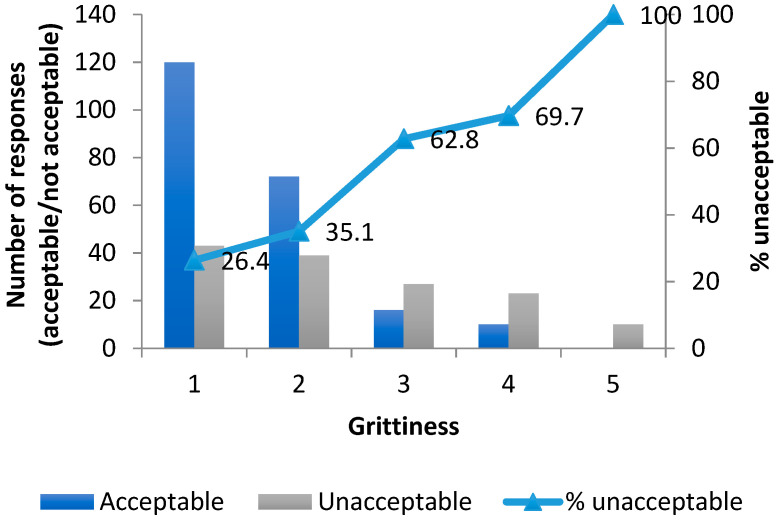
Cumulative frequency of unacceptability as a medicine with grittiness for all 360 evaluations.

When the acceptability as a medicine dataset of 360 samples (20 volunteers, 18 samples per volunteer) was analysed by grittiness score (1–5), and summarized as percentage acceptable/unacceptable at each grittiness level, it was found that there was an overall association between unacceptability and grittiness. The proportions of the samples found unacceptable as a medicine increased consistently across the range of evaluated grittiness score (Figure 6).

The second approach used a quantitative statistical method, which found that the odds of a sample being evaluated as acceptable as a medicine was four times greater for non-gritty samples than for gritty samples. The logistic regression model showed a good fit with X^2^ (2) of 332.1 and a *p* value < 0.001. The model explained 81.87% (Nagelkerke R square) of the variance in acceptability with 91.9% accuracy.

The regression equation was:

Log (*p*/1 − *p*) = −4.397 + 0.095rating + 1.420grittiness (1)

(where rating is the taste or aversion score, and grittiness is the transformed scale in this case referring to not gritty samples).

### 3.4. Qualitative Comments

A number of qualitative comments mentioned the smell of the infant formula milk and an “after-taste” on expectoration of the infant formula milk dispersed formulations as “turn-offs”. A couple of these comments were:

“The taste is not bad but after splitting (sic) it out, the lefting (sic) smell makes me a little bit unhappy”, and “The aversiveness was from the taste of the artificial milk rather than perceiving any taste of drug. The taste of the sample itself was not bad when it was in the mouth, but the smell and after-taste were off-putting. It smelt almost like milk that had slightly gone off. There was no grittiness”.

While these, in addition to the assessment of taste *per se*, are important considerations in medicine design, they are in the context of this investigation related to the reconstitution vehicle and not the pharmaceutical form. It should also be noted that a child’s perception of taste is different to adults; perhaps of importance, this may especially be the case with breast milk/infant formula milk [27,28].

The zinc formulations were described as astringent and gritty. Those dispersed in infant formula milk were described as having an unpleasant after-taste. Unpleasantness was reported to increase with the residence time of the sample in the mouth, and on expectorating or spitting-out. The taste of the infant formula milk itself was variable and included both comments related to pleasant and unpleasant.

## 4. Discussion

Medicines designed for young children should be easy to administer and dose as well as ideally be palatable, without being mistaken for confectionary [7]. During the medicine development process, human taste panels remain the “gold standard” method for taste assessment and were, therefore, used in this study [29].

In this palatability assessment study, which, to our knowledge, is the first of its kind with milk, we investigated the ability of formula milk to taste-mask two selected APIs and for its potential to improve the palatability of two purposively-designed dispersible tablet platforms. In addition, we conducted a sensory evaluation of the platforms themselves.

The placebo control blends yielded very low aversion values on reconstitution as aqueous suspensions. That the platform formulations did not differ in terms of aversion scores, was possibly due to the identical proportion of the polyols mannitol and lactose contained, as both polyols are classified as “mildly sweetening” [30]. The similarity in taste of the two formulation platforms suggests the potential for a lead and back-up placebo platform approach as a starting point to paediatric formulation development work. Prior knowledge of API physicochemical properties, for example, the Maillard reaction between drug substances with primary amine functional group and lactose [30], can drive the decision as to which platform is chosen initially when developing a new paediatric product.

The formulation platforms developed have been successfully tableted on a single station Manesty F-press [11] and have potential to be scaled-up to support paediatric drug products to support not only low resource settings but also to be used as a global age-appropriate product, minimising supply chain issues and maximising access to medicines for a particularly vulnerable patient cohort. However, the corresponding powder mix was used as it was not possible within the remit of the study to produce tablets of a quality for human consumption.

While there were indications that the aversion score could be affected by formulation platform, API and reconstitution vehicle, the only statistically different combination of the three variables was found when comparing the potential for infant formula milk to mask the taste of mannitol (T2)/zinc sulfate formulations when compared to reconstitution in water. The reason for the significantly lower aversion scores in the mannitol (T2)/zinc/infant formula milk formulation compared to the mannitol (T2)/zinc/water formulation is unclear. Given the differences in formulation composition of the lactose (T1) and mannitol (T2) platforms, it is possible that a complex physical interaction is occurring between zinc sulfate, one or more of the ingredients in Aptamil^®^ 1, and one or more of the following excipients in the T2 formulation: mannitol, crospovidone and sodium stearyl fumarate (SSF), including any viscosity effect of the vehicle. Given the low weight percentage at which the disintegrant (crospovidone) and lubricant (SSF) are present in the T2 formulation, the potential taste masking phenomenon observed with the T2 formulation dispersion is more likely a result of the combined presence of the filler, mannitol, and the infant formula milk dispersion medium, including possible solubilization [31]. In addition, it is possible that the cooling effect that can be elicited by mannitol might have contributed [32]. However, it should be noted that the two APIs have different “types” of aversiveness. Compared to zinc, which has been described as metallic rather than bitter, though also assessed as astringent, paracetamol is noticeably bitter [33]. This difference means that the taste of paracetamol is more discernible and, thus, inherently more difficult to mask.

To resolve this complex interaction, which is further complicated by the multi-component composition of infant formula milk, would involve a large and complex design of experiments (DoE) with many samples. This was outside the scope of the present study to determine. Human taste panel evaluation, which involves a lengthy ethics application and preparation process, as well as a commitment on the part of the volunteers to attend all sessions, does not lend itself well as an endpoint or assay for such a DoE. In recent years, in vivo methodologies have been developed and validated as alternative assays to in vitro techniques such as the electronic tongue [16,34,35,36]. Such minimally-invasive novel methodologies offer the potential to generate more relevant in vivo taste data than is offered by in vitro techniques, and may be of use as a formulation screening tool prior to progressing to human taste panels with two or three lead formulations to generate the most informative data (acknowledging differences in perception of taste in adults and children). 

Over the last few decades, there has been an understandable increase in research into taste masking and assessment of pharmaceutical formulations driven by recently introduced US and EU legislations regarding the development of age-appropriate medicines for children [6]. A contributory driver to this is an acknowledgement that taste is a major factor affecting medicine compliance in children [37]. 

An outcome of the analysis of the current work is the establishment of a relationship, in a limited number of volunteers, between grittiness of an administered formulation and its acceptability/unacceptability. This result conforms with other studies assessing the effect of grittiness (particle size) on the palatability of a medicine, which also showed that grittiness impacts palatability [29,34,38,39]. This is a strength of the study.

The difference in grittiness between the lactose-based and mannitol-based placebos may be related to the content of crospovidone in the mannitol-based formulation. Included at 5% to promote disintegration, it has an average particle size range of 110–140 µm, in the grade used. Thus, the mannitol platforms contained at least 5% more content of an insoluble particle. This may be the reason why its placebos were assessed as gritty by some participants, compared to the lactose-based formulations, as the particle size threshold for eliciting grittiness is estimated by different researchers, with values ranging from 70 to 300 µm [40,41,42]. 

In addition to particle size, other factors affecting the perception of grittiness are the volume fraction of the insoluble ingredients, the viscosity of the dispersion medium, shape and hardness of the particles [38]. Increasing the viscosity of the dispersion medium can decrease the perception of grittiness, and rough hard particles can be perceived as gritty at particle sizes smaller than for smooth, soft particles. The fat content in milk can also adsorb or dissolve poorly-soluble bitter APIs or coat the taste buds to “blur” taste. However, more work is needed to better determine the relationship between the grittiness of a medicine and its “acceptability”. Furthermore, more work is needed to better understand when and why taste masking can be achieved by reconstitution of age-appropriate medicines in milk, and possibly other types of milk (formula, animal, vegetable, human). These different types of milk would have different tastes. It should also be noted that milk-fed infants have a very different taste perception to adults and are more sensitive to bitter taste. 

The incorporation of small amounts of an intense sweetener at a level acceptable for younger children could boost the humble taste masking effect seen in this study. This can be considered in further development of the platforms. Such work should take into consideration safety concerns with some excipients used for taste masking [3].

One limitation of this work was that the homogeneity of the powder blends was only assessed visually. However, homogeneity was affected/attempted by gentle rotation of the sample tubes for 10 s before presentation to the subjects. 

## 5. Conclusions

This study explored the palatability of two novel dispersible tablet platforms containing zinc and paracetamol dispersed in infant formula milk. 

The mannitol- and lactose-based placebo platforms are not aversive in the taste panel used. They, therefore, show potential to be used as a starting point to support the development of fast-dissolving solid oral dosage forms that can be adapted for drug delivery to all paediatric populations, adolescents to neonates, as clinical development progresses into younger children, in accordance with a Paediatric Investigation Plan.

Use of the platforms, in line with the WHO flexible solid dosage forms proposal, could streamline and expedite the drug development process by facilitating a fit-for-purpose/exploratory early phase PK formulation that can be used in children in all paediatric age ranges, thus reducing formulation bridging risk throughout the clinical development.

As the formulation platforms use only GRAS excipients, which are suitable for onward direct compression into tablets of varying size and drug potency, the platforms also represent a strategic development route to a globally commercialisable formulation.

The infant formula milk successfully masked the taste of zinc sulfate in the mannitol-based (T2) formulation but not in the lactose-based (T1) formulation. The infant formula milk did not, however, mask the taste of paracetamol in either formulation. 

## Figures and Tables

**Table 1:** Composition of the two platform blends assessed in the sensory analysis. The blends (lactose-based, T1, and mannitol-based, T2) were reconstituted in water and infant formula milk (batch sizes; samples contained 300 mg of each formulation) containing: (a) zinc, (b) paracetamol and (c) no API (placebo).

Dispersible Tablet Platform			a			b			c	
			%	g *		%	g *		%	g *
**Lactose-based, T1**	Zinc	ZnSO_4_·7H_2_O	26.66	87.98	Paracetamol	33.15	120.00	Placebo	0	0
		Lactose	57.34	189.22		52.27	189.22		78.19	189.22
		Microcrystalline cellulose	10	33.00		9.12	33.00		13.64	33.00
		Sodium starch glycollate	3	9.90		2.73	9.90		4.09	9.90
		Sodium croscarmellose	2	6.60		1.82	6.60		2.73	6.60
		Magnesium stearate	1	3.30		0.92	3.30		1.36	3.30
		total	100	330		100	362		100	242
**Mannitol-based, T2**	Zinc	ZnSO_4_·7H_2_O	26.66	87.98	Paracetamol	33.15	120.00	Placebo	0	0
		Mannitol	57.34	189.22		52.27	189.22		78.18	189.22
		Microcrystalline cellulose	10	33.00		9.12	33.00		13.64	33.00
		Crospovidone	5	16.50		4.55	16.50		6.82	16.50
		Sodium stearyl fumarate	1	3.30		0.92	3.30		1.36	3.30
		total	100	330		100.01	362		100	242

Footnotes: S3, S4. * Totals are rounded up to the nearest whole number.

**Table 2:** P-values for the difference in mean taste-scores in the assessment of the taste-masking ability of infant formula milk. Note: T1 is the lactose platform, and T2 is the mannitol platform. Para = paracetamol.

Formulation Pairs	N	Mean	S. D	*p* Value
T1paramilk	20	77.90	18.94	0.344
T1parawater	20	73.70	18.28	
T2paramilk	20	81.20	17.74	0.121
T2parawater	20	72.70	20.90	
T1zincmilk	20	48.60	30.23	0.263
T1zincwater	20	56.75	32.20	
T2zincmilk	20	43.95	28.40	0.015
T2zincwater	20	60.15	31.33	

## Data Availability

All relevant data included in the manuscript.

## Supplementary Materials

The following supporting information can be downloaded at: https://www.mdpi.com/article/10.3390/pharmaceutics14020420/s1. Supplementary Materials: Evaluating the Taste Masking Ability of Two Novel Dispersible Tablet Platforms Containing Zinc Sulfate and Paracetamol Reconstituted in a Breast Milk Substitute [11,16,43,44,45].
