# Supplementary material for: Evaluating the Taste Masking Ability of Two Novel Dispersible Tablet Platforms Containing Zinc Sulfate and Paracetamol Reconstituted in a Breast Milk Substitute
Source: Pharmaceutics. 2022 Feb 15;14(2):420. doi: 10.3390/pharmaceutics14020420 (PMC8878340; doi:10.3390/pharmaceutics14020420)
Supplement: Supplementary file 1 [file pharmaceutics-14-00420-s001.zip › pharmaceutics-1514051-supplementary.pdf]

pharmaceutics

Supplementary Materials: Evaluating the Taste Masking Ability of Two Novel Dispersible Tablet Platforms Containing Zinc Sulfate and Paracetamol Reconstituted in a Breast Milk Substitute

Samuel Orubu, Richard A Kendall, Yucheng Sheng and Catherine Tuleu

S1. Aptamil

This palatability study evaluated only liquid Aptamil. In an earlier study, we had evaluated a range of milk/infant formula products such as commercially available in the supermarkets in one developed and one selected developing country yet covering a range of different compositions [1]. In the published study, we had compared four different milk types as well as made tablets from them that were reconstituted in water [2]. Thus, we had robustly investigated, using a design of experiment approach, several milk and infant formula types for a range of properties from compatibility to powder flow to direct compression manufacturability (as tablets) to tablet evaluation, including reconstitution in water.

In this study, the focus was on evaluating both the palatability of these novel dispersible tablet powder blends and the ability of infant formula milk to provide taste-masking of two selected APIs, in the first study to have assessed this. The rationale for choosing liquid Aptamil was the on-the-shelf availability in the UK of a non-specialized infant formula suitable for use in the age group of 0-6 months. Thus, the choice was simply suitability for the purpose of the research as it met our criteria. There is no connection whatsoever between the manufacturers of Aptamil and this study. The tablet blends evaluated in the present study have been comprehensively investigated as tablet formulations in other studies [2,3].

S2. Sieving

The work described is a pre-formulation clinical study. We used the corresponding tablet blends. There was no tablet production process. Sieving was performed using a single 500-micron sieve after powder blending to ensure that the blends were below the acceptable particle size for of 750 microns for dispersible tablets as specified in the British Pharmacopoeia – even though tablets were not produced in this study. This was a gentle "screening" sieving, not of the magnitude to induce segregation as may occur for tableting.

S3. Differences between formulations and rationale

The differences represent a scoping/screening attempt using different fillers (in this case, mannitol and lactose) with different compression properties, and commercial availability from the perspective of a Low- and Middle-Income country. The goal was to identify lead and back-up formulations that could both be used for the direct compression production of dispersible tablets in a resource-limited country. The variations in quantities are based on the compression experiments to obtain tablets that met the British Pharmacopoeial specification for dispersible tablets (disintegration time <1 minute) with suitable handling properties (tensile strength) [1,2]. Generally, a back up to lactose is needed, for reasons of any potential API incompatibility issues such as its well-known reaction with primary amines.

S4. Compatibility

The potential interaction among these excipients have been investigated using standard pre-formulation Differential Scanning Calorimetry, Fourier Transform Infra-Red Spectroscopy and XR diffraction studies as reported in earlier studies [1,2].

S5. Number of participants in the taste panel

From a statistical standpoint, 20 participants present a sample size considered suitable for a pilot study. However, considering that the study assessed a total of 360 taste samples, we think this number was sufficient for a study of this size. Additionally, 20 participants had also been used in a similar study [4].

S6. Choice of human taste panel

Our group at UCL has conducted several of these in vivo methodologies (and in vitro, as well). Of all, the human taste panel, as we employed in this study, remains the most established and this informed its choice in this study. For example, while the rat brief aversion taste assessment method developed by other researchers in our group [5] has been validated for bitterness, it is yet to be validated for assessing grittiness. The human taste panel was considered, given its relative robustness, the more suitable for this study.

S7. Use of an adult taste panel

The direct use of children as taste panels for paediatric medicines is not common practice because of ethical concerns and lack of reliability of outputs. Adult volunteers represent an acceptable proxy [4].

References

1. Orubu, S.E.F.; Hobson, N.J.; Basit, A.W.; Tuleu, C. The Milky Way: paediatric milk-based dispersible tablets prepared by direct compression—A proof-of-concept study. *J. Pharm. Pharmacol.* **2017**, *69*, 417–431, doi:10.1111/jphp.12570.
2. Orubu, E.S. Flexible Solid Oral Dosage (FSOD) Forms as the Preferred Formulations for Children—UCL Discovery. Available online: https://discovery.ucl.ac.uk/id/eprint/1476962/ (accessed on 28 September 2020).
3. Scheuerle, R.L.; Bruggraber, S.F.A.; Gerrard, S.E.; Kendall, R.A.; Tuleu, C.; Slater, N.K.H. Characterisation of zinc delivery from a nipple shield delivery system using a breastfeeding simulation apparatus. *PLoS ONE* **2017**, *12*, e0171624, doi:10.1371/journal.pone.0171624.
4. Orlu-Gul, M.; Fisco, G.; Parmar, D.; Gill, H.; Tuleu, C. A new reconstitutable oral paediatric hydrocortisone solution containing hydroxypropyl-β-cyclodextrin. *Drug Dev. Ind. Pharm.* **2012**, *39*, 1028–1036, doi:10.3109/03639045.2012.696654.
5. Soto, J.; Keeley, A.; Keating, A.V.; Mohamed-Ahmed, A.H.; Sheng, Y.; Winzenburg, G.; Turner, R.; Desset-Brèthes, S.; Orlu, M.; Tuleu, C. Rats can predict aversiveness of Active Pharmaceutical Ingredients. *Eur. J. Pharm. Biopharm.* **2018**, *133*, 77–84, doi:10.1016/j.ejpb.2018.09.027.
